# Acute and Chronic Effects of Stretching on Running Economy: A Systematic Review with Meta-Analysis

**DOI:** 10.1186/s40798-025-00859-0

**Published:** 2025-05-30

**Authors:** Konstantin Warneke, Maximilian Zechner, Stanislav D. Siegel, Daniel Jochum, Leefke Brunssen, Andreas Konrad

**Affiliations:** 1 Institute of Human Movement Science, Sport and Health, University of Graz, Graz, Austria; 2 Department for Human Motion Science and Exercise Physiology, University of Jena, Jena, Germany; 3 Department of Health Sciences and Technology, ETH Zurich, Zurich, 8092 Switzerland; 4 Department of Sport Science, Bielefeld University, Bielefeld, Germany

## Abstract

**Background:**

Running economy (RE) determines the performance of endurance athletes. While stretching has been practised for decades, and is still one common integral component of warm-up routine, muscle stretching is also associated with decreased stiffness. For RE energy storage in the tendons which is accompanied with stiffness is of crucial importance. In turn, avoidance of pre-running stretching was frequently recommended. Although some studies supported this recommendation, the evidence is controversial. Nevertheless, yet, no systematic review on the effects of stretching on RE with effect size (ES) quantification was performed. Consequently, with this systematic review with meta-analysis, we aim to provide the first overview on this topic.

**Methods:**

In adherence to PRISMA 2020 guidelines, we meta-analyzed effect sizes from three databases using PICOS guidelines on stretching effects on RE in healthy participants using robust variance estimation. Heterogeneity was reduced using subgroup analyses while meta-regression evaluated whether running velocity potentially moderates results. Risk of Bias was assessed using the PEDro scale, certainty of evidence was classified via GRADE working group criteria. The study protocol was registered in Open Science Framework 10.17605/OSF.IO/MA8D4).

**Results:**

Overall, low certainty of evidence pooled from 15 studies with a total of 181 participants indicated that stretching did not significantly moderate RE acutely (*p* = 0.21–0.65), neither in general, nor were there any stretching types (dynamic, static and proprioceptive neuromuscular facilitation) that affected this result. Due to the limited number of chronic studies found in the literature, long-term stretching effects were exclusively evaluated qualitatively. Meaningful heterogeneity and reduced methodological quality (PEDro Score: 4.88, fair) contributed to certainty of evidence downgrading.

**Conclusions:**

In contrast to common beliefs that stretching decreased stiffness parameters and would therefore hamper RE, current evidence does not support any effect of stretching on RE in running athletes. However, several flaws such as no investigation of the underlying mechanisms (e.g., stiffness), small sample sizes, determining RE at different velocities, and the implementation of unreasonable stretching durations strongly biased conclusions. Especially on chronic effects there is a large demand for improved evidence, including underlying mechanisms investigation. Yet, it seems unreasonable to avoid pre-running stretching to prevent RE decreases.

**Supplementary Information:**

The online version contains supplementary material available at 10.1186/s40798-025-00859-0.

## Key Points

• In general, 15 acute and 2 chronic studies were identified exploring stretching effects on running economy, using static, dynamic or proprioceptive neuromuscular facilitation stretching.

• There was low certainty of evidence indicating no significant effect of any type of stretching on running economy acutely (*p* = 0.21–0.65), while the small number of chronic studies prohibited meta-analyzing chronic effects.

• Methodological limitations, including small sample sizes, heterogeneous protocols, and lack of mechanistic insights (e.g. muscle stiffness), suggest the need for higher-quality studies, particularly on chronic stretching effects.

## Background

On the 8th of October 2023, Kelvin Kiptum broke the men’s marathon world record again, setting a time of 2 h and 35 s. With this performance, he continued the unmatched phenomenon of Kenyan distance runners’ domination at global events [1]. The goal in running competitions is always to cover a distance in the shortest possible time, whereby the athletes’ physiological and anatomical performance determinants vary depending on the respective distance. In contrast to sprinters, whose performance is determined by their ability to generate maximum impulses, thus converting a large amount of energy within a relatively short time window [2], the long-distance runner seeks the most economical movement pattern [3]. This crucial performance parameter is known as running economy (RE) [4], which is defined as oxygen consumption at a given running pace (in ml*min^− 1^*kg^− 1^) [5]. The longer the distance, the more important RE becomes to consume the lowest amount of energy while maintaining the highest velocity possible [4].

As a complex multifactorial construct, Barnes and Kilding [3] described RE as being influenced by a plethora of factors, involving metabolic and cardiovascular, but also neuromuscular and biomechanical measures. Among these factors, the athlete’s anatomy, such as the Achilles tendon length, the muscle/tendon torque arms [6–9], and structural properties such as tendon and muscle stiffness [10, 11] and fascicle lengths [6], is related to a more economical way to move. Accordingly, energy storage and generation in the stretch-shortening cycle appear to be dependent on the properties of the muscle-tendon unit (MTU) [10, 12]. Arampatzis et al. [13] showed that economical runners generate, on the one hand, higher contractile forces, and on the other hand, show higher normalized Achilles tendon stiffness, which is defined as the relationship between tendon force and tendon strain. Meanwhile, they also noted that a more compliant quadriceps MTU is beneficial. These attributes can result in a decreased active muscle volume involved in running by simultaneously increasing the force generation at low-level forces [13].

As the energy cost caused by muscular activity seems to crucially determine the overall energy [14] and oxygen consumption in submaximal runs [15], attempts have been made to improve the spring-like properties of the Achilles tendon via exercise [10, 16]. One suggested approach to increase tendon stiffness is strength training, even though the study effects seem contradictory. While a 14-week exercise program increased the Achilles tendon stiffness and decreased the rate of oxygen consumption and energy cost by 4% [17], 12 weeks of 4 × 20 s isometric training of the calf muscles affected neither tendon stiffness nor RE [18]. The current systematic reviews with meta-analysis considered different types of training and showed that high resistance training has a positive influence on RE, while submaximal methods do not [19–21]. In this manner, the higher strain of resistance training is correlated with greater increases in stiffness [22].

Another common intervention with the potential to affect muscle-tendon properties is stretching [23–26]. In contrast to resistance training, a single stretching exercise [25], as well as stretch training for several weeks [23, 24], is mostly associated with decreased stiffness in the muscle-tendon tissue, besides other mechanisms such as neurological changes (e.g., pain perception) [27, 28]. Due to the speculated positive influence on muscle-tendon injuries [29], the inclusion of stretching in warm-up programs for running athletes is stated frequently in the literature [30]. However, the acute and chronic effects of passive [31], static [32] or dynamic [32–35] stretching on RE have been a subject of controversy in the literature, with negative [31, 33], positive [32, 34], and no effects [35] being reported. It appears that there is an optimal degree of stiffness and flexibility for maximizing RE [3]. While there have been several reviews on the effects of resistance training on RE, to date, only one scoping review, but no systematic review was performed on stretching effects on RE. Only one scoping review [36] has drawn conclusions based on qualitative individual study results and favored dynamic stretching over static stretching in warm-up routines for runners.

Due to its possible impact on muscle-tendon properties and the demand for high-level evidence, this systematic review’s objective is to pool the effect sizes (ES) from the available evidence to establish the potential harm or benefit on RE of movement preparations using stretching (i.e., warm-up effects) and whether the available literature suggests that the implementation of regular stretching sessions can induce chronic responses in RE.

## Methods

Adhering to the PRISMA 2020 (Preferred Reporting Items for Systematic Reviews and Meta-Analyses) guidelines, we conducted a systematic review with multilevel meta-analysis (a checklist is attached to the Supplementary Material). We considered ethical publishing standards [37] and registered the study in the Open Science Framework (registration 10.17605/OSF.IO/MA8D4). Registration was completed after the preliminary search to identify the proper study design (meta-analysis or qualitative systematic review depending on number of eligible studies/number of studies with comparable study design).

### Literature Search

#### Eligibility criteria

Only studies in English language published in peer-reviewed journals were considered. According to “Participants, Intervention, Control, Outcome and Study design” (PICOS) guidelines, studies were considered as eligible for inclusion if they:

1. P: Included healthy adults (group mean ≥ 18 years of age) participants without restriction by sex or their training status.
2. I: Performed any type of (unloaded) stretching (e.g., static, dynamic/ballistic, proprioceptive neuromuscular facilitation (PNF)). The different types of stretching were defined in accordance with Warneke and Lohmann [38] and Behm [39]. Therefore, stretching was considered static if the muscle was lengthened until the onset of a stretch sensation or to the point of discomfort. By definition, this position is to be held and can be performed passively via partner, external weight, or tool, or actively via antagonist contraction. PNF stretching consists of a (sub)-maximal voluntary contraction to a stretching bout with or without antagonist contraction, while dynamic stretching is defined as controlled back-and-forth movement at the end range of motion (ROM), with ballistic stretching as a sub-category with less controlled, bouncing movements [40].
3. C: In the control condition, participants either performed a general warm-up (e.g., jogging) or rested. The control condition was defined as performing the same warm-up exercises as in the intervention condition, except for the stretching component.
4. O: Assessed acute or chronic effects on RE and related parameters (oxygen consumption, energy cost of running), whereby effects were considered as chronic when more than one intervention session was conducted and pre- and post-test were divided by at least two weeks.
5. S: Used Randomized or non-randomized Controlled Trials with pre-post or post-only Comparisons.

#### Information sources, search strategy and selection process

Two authors (SDS and LB) independently conducted the search using three databases: MEDLINE/PubMed, Web of Science (Core Collection), and Scopus (inception to April 2024, updated January 2025). This was supplemented by manual search in Google Scholar and a review of the reference lists of identified articles (KW).

The following search terms were:

((((((((“runn*“[Title/Abstract]) OR “aerob*“[Title/Abstract]) OR “anaerob*“[Title/Abstract]) OR “running economy“[Title/Abstract]) OR “running performance“[Title/Abstract]) OR VO2[Title/Abstract]) OR “oxygen uptake“[Title/Abstract]) OR “energy cost“[Title/Abstract]) AND ((“stretch*“[Title/Abstract]) OR “flexibility training“[Title/Abstract])

Studies were excluded if they assessed stretching effects in unhealthy populations/patients or children. Furthermore, direct RE or related parameters of interest (oxygen consumption, energy cost of running) had to be provided. If the authors did not provide means and standard deviations, and these could not be imputed from a graphic, we contacted the authors via e-mail or ResearchGate. However, if the authors did not respond, we excluded the study from the review.

#### Data items

Our outcome of interest was RE [4], which is defined as oxygen consumption (VO2) at a given workload or velocity [5]. Therefore, we extracted also the intensity at which VO2 was tested as well as the duration of the test. Further, we included several related parameters, for instance, but not limited to oxygen consumption at several intensities or energy consumption in kilocalories (kcal) [41] or kilojoules per kilogram bodyweight per minute (kJ/min) [42] at a given workload.

Other extracted variables included participants characteristics (age, weight, height) as well as their running training status (as defined by the study, e.g. recreationally trained; number of training sessions per week). Further, intervention characteristics of stretching included type of stretching as well as exercise descriptions (e.g. targeted muscle, number of sets, reps, time per rep, inter-set rest, overall volume, stretching intensity). These are provided in Table 1.

**Table 1:** Study characteristics, intervention protocols, and key outcomes

Study	Participants	Methods	Outcomes	Results
Allison et al. [51]	*n* = 10 (m: *n* = 10), age: 25 ± 5 yrs., height: 176 ± 4 cm, mass: 72,2 ± 0.3 kg. Healthy adults. Crossover design with random allocation (2 groups each *n* = 10). Male runners who do > 5 h of regular endurance training per week	Interventions: Static stretching and non-intervened CG. Muscle(s): knee extensors, hip flexors, knee flexors and plantar flexors. IG protocol: unilateral stretching of each leg for 3 × 40 s at the point of mild discomfort with eight exercises. Intervention period: 4 sessions within 10 days. CG protocol: No intervention.	70% Sub-maximal running performance and peak oxygen uptake, pulmonary gas exchange, RE test, running stride, sit and reach range of motion, isometric strength, countermovement jump	RE test: IG: VO_2_ (ml/kg/min) Pre: 41.4 ± 6.4, Post: 41.7 ± 6.5 EE (kJ/min) Pre: 61.4 ± 8.3, Post: 61.8 ± 8.8, VE (l/min) Pre: 66.4 ± 2.1, Post: 67.9 ± 2.7 RER Pre: 0.92 ± 0.02, Post: 0.92 ± 0.03, HR (bpm) Pre: 144 ± 4, Post: 45 ± 6. CG: VO_2_ Pre: 41.3 ± 5.9, Post: 41.3 ± 6.0, EE Pre: 61.1 ± 8.6, Post: 61.2 ± 8.4, VE Pre: 66.2 ± 2.8, Post: 68.5 ± 2.6, RER Pre: 0.91 ± 0.03, Post: 0.90 ± 0.03, HR Pre: 144 ± 4, Post: 145 ± 6. Running stride: IG: stride frequency (strides/s) Pre: 1.36 ± 0.06, Post: 1.35 ± 0.06, stride length (m) Pre: 2.27 ± 0.41, Post: 2.29 ± 0.43. CG: stride frequency, Pre: 1.36 ± 0.08, Post: 1.36 ± 0.07, stride length Pre: 2.27 ± 0.38, Post: 2.27 ± 0.44. Sit and reach score (cm): IG: Pre: 0.4 ± 2.6, Post: 3.1 ± 2.5,
Damasceno et al. [42]	characteristics: *n* = 11 (m: *n* = 11), age: 35.7 ± 6.1 yrs., height: 176 ± 0.08 cm, mass: 79.7 ± 11,3 kg. Healthy adults training status: Recreationally trained long-distance runners (30 km/week) with no previous experience in strength or plyometric training. Crossover design with random allocation (2 groups with each *n* = 11).	Static: 5 unassisted exercises (straight-leg stand and toe touch, standing quadriceps stretching, hamstrings and back stretching, hurdler’s stretching, standing calf stretching) and 2 assisted exercises (quadriceps and hip stretching, and thigh stretching) were performed for 3 × 30 s. 5 sessions within 3 weeks. control: no intervention Muscle(s): lower limbs.	Laboratory tests: anthropometric measurements, maximal incremental test, constant-speed test. Field tests: 3-km running test, sit and reach test, drop jump test.	12 km/h RE (ml/kg/min): CG: 41.3 ± 2.8, IG: 40.4 ± 3.0. Caloric unit cost (kcal/kg/min): CG: 1.03 ± 0.07, IG: 1.00 ± 0.08. iEMG_VM_ (µV): CG: 60 ± 21, IG: 64 ± 23. iEMG_GA_ (µV):: CG:77 ± 27, IG: 95.3 ± 38. iEMG_BF_: CG: 73 ± 26, IG: 94 ± 31. Contact time (ms): CG: 256 ± 26, IG: 250 ± 32. Flight time (ms): CG: 443 ± 42, IG: 453 ± 37. Stride time (ms): CG: 697 ± 41, IG: 710 ± 41. iEMG_BF_ was significantly higher in SS condition. The stride time was significantly longer in SS condition.
Faelli et al. [32]	*n* = 8 (m: *n* = 8), age: 36 ± 11.51 yrs., height: 176.53 ± 6.36 cm, mass: 71.99 ± 9.65 kg. Healthy adults. Crossover design with random allocation (3 groups each *n* = 8). Recreational male runners with a training volume of about 15 km/week.	Interventions: Static stretching, dynamic stretching and non-intervened CG. Muscle(s): Quadriceps, hamstrings, hip flexors, hip adductors and gluteals. IG1 protocol: Unilateral static stretching 1 × 30 s. IG2 protocol: Unilateral dynamic stretching 1 × 30 s. CG protocol: No intervention. 4 sessions (1 session per week).	Physiological and metabolic parameters, running performance parameters, RE rating of perceived exertion.	VO_2rest_ (ml/kg/min): IG1: 5.81 ± 0.32 IG2: 5.85 ± 0.32 CG: 6.08 ± 0.26 VO_2_ (70% VO_2max_): IG1: 24.70 [24.15, 25.25] IG2: 23.95 [23.05, 24.75] CG: 29.70 [29.12, 30.58] VO_2max_: IG1: 50.15 [48.20, 51.58] IG2: 51.15 [50.13, 52.90] CG: 49.70 [48.30, 51.50] HR (bpm): IG1: 176.63 ± 8.75 IG2: 177.63 ± 10.47 CG: 176.50 ± 10.94 La (mmol/l): IG1: 15.09 ± 2.43 IG2: 15.18 ± 2.79 CG: 14.22 ± 3.32 Time to exhaustion (s): IG1: 161.63 ± 42.16 IG2: 166.63 ± 45.00 CG: 164.38 ± 36.43 Total running distance (m): IG1: 666.88 ± 171.84 IG2: 676.38 ± 163.16 CG: 669.88 ± 142.84 70% VO_2max_ RE (ml/kg/min): IG1: 19.05 [17.7, 20.3] IG2: 18.20 [16.85, 19.90] CG: 23.40 [22.83, 24.20]. Significant improvement in RE and a reduction in the perception of exertion in both IGs compared to CG.
Godges et al. [52]	*n* = 25 (m = 25) healthy, athletic, male college students (Mean age = 21 years, Mean weight = 75 kg, Mean height = 172 cm). criterion for admission to the study was the presence of limited hip extension ROM as measured by a modified Thomas test.	control group (*n* = 7) No intervention. hip extension stretching group (*n* = 9) Static stretching applied at the end of available hip extension ROM, using a protocol based on the intensity and duration of sustained passive stretch intensity = 15% of subjects body weight duration: 2 min interset pause: 2 min sets: 3 2x per week trunk flexor exercise group (*n* = 9). Followed a leg-lowering exercise progression with an emphasis on stabilization of the pelvis by the trunk flexors. 5 min 2x daily for 3 weeks	40% VO_2max_ (108 m/min) walking economy, 80% (200 m/min) VO_2max_ RE trunk flexor muscle performance (Goniometer) right hip extension ROM (Goniometer) left hip extension ROM (Goniometer)	Hip Extension ROM (°): Control Group: right side: pre: -15.8 ± 10.4, post: -18.4 ± 9.2 left side: pre: -17 ± 6.6, post: -17.4 ± 8.6 Stretching Group: right side: pre: -20.4 ± 4.2, post: -8.3 ± 6.2 left side: pre: -16.8 ± 4.2, post: -7 ± 6.3 Trunk Exercise Group: right side: pre: -21.1 ± 4.0, post: -23.6 ± 6.3 left side: pre: -19.3 ± 4.2, post: -21.1 ± 4 Walking Economy (mL · kg^-1 · min^-1): Control Group: pre: 17.9 ± 1.2, post: 16.4 ± 2.2 Hip Stretching Group: pre: 17.9 ± 1.3, post: 17.8 ± 0.9 Trunk Exercise Group: pre: 17.4 ± 1.5, post: 16.1 ± 2.8 RE (mL · kg^− 1^ · min^-1): Control Group: pre: 39.3 ± 2.8, post: 38.0 ± 2.7 Hip Stretching Group: pre: 39.5 ± 1.7, post: 38.7 ± 1.7 Trunk Exercise Group: pre: 38.8 ± 2.8, post: 38.5 ± 3.6
Hayes & Walker [53]	*n* = 7 (m: *n* = 7), age: 32.5 ± 7.7 yrs., height: 175 ± 8,8 cm, mass: 67.8 ± 8.6 kg. Healthy adults. Crossover design with random allocation (4 groups each *n* = 7). Competitive male middle and long-distance runners with a training volume about 68 km per week.	Interventions: Static stretching, controlled velocity dynamic stretching, progressive stretching and non-intervened CG. Muscle(s): Major muscle groups used during running. IG1 protocol: Unilateral static stretching (standing quadriceps stretch, standing calf stretch, lunge, seated hamstring stretch and seated gluteal stretch) 2 × 30 s. IG2 protocol: Unilateral controlled velocity dynamic stretching (same exercises as SS) 2 × 30s. IG3 protocol: Unilateral progressive static stretching (same muscles as SS) 2 × 30 s. CG protocol: No intervention.	Measurement of lactate threshold and VO_2max_, sit and reach test, measurement of steady state VO_2_ and RE, analyses of expired gases	RE (ml/kg/km): IG1: 202 ± 18.4 IG2: 198.2 ± 18.7 IG3: 196.3 ± 17.4 CG: 199 ± 19.1 Sit and reach: All intervention groups show a significant difference between pre- and post stretching. No significant difference in CG.
Konrad et al. [54]	*n* = 18 (m: *n* = 18), age: 30 ± 6.1 yrs., height: 182.5 ± 4.6 cm, mass: 75.4 ± 7.7 kg. Healthy adults. Crossover design with random allocation (3 groups each *n* = 18). 18 trained runners/triathletes with a training volume of at least 30 km per week for at least 2 years.	Interventions: PNF stretching + post-stretching dynamic activities (PSA) and non stretching + PSA CG. Muscle(s): quadriceps and triceps surae IG1 protocol: 4 × 15 s unilateral PNF stretching exercise of the triceps surae + PSA IG2 protocol: 4 × 15 s unilateral PNF stretching exercise of the quadriceps + PSA. CG protocol: No stretching + PSA. 5 sessions within 2 weeks.	RE (70% VO_2max_), ground contact time, stride length and stride frequency.	Oxygen consumption (L/min): IG1: 3.11 ± 0.54 IG2: 3.14 ± 0.58 CG: 3.05 ± 0.49 Ground contact time (s): IG1: 0.287 ± 0.034 IG2: 0.286 ± 0.031 CG: 0.290 ± 0.032 Stride length (cm): IG1: 232.7 ± 24.8 IG2: 233.7 ± 26.3 CG: 232.6 ± 25 Stride frequency (stride/s): IG1: 1.35 ± 0.07 IG2: 1.34 ± 0.07 CG: 1.35 ± 0.07
Maior et al. [31]	*n* = 8 (m: *n* = 8), age: 24.6 ± 5.5 yrs., height: 179 ± 4.1 cm, mass: 78.1 ± 3.4 kg. Healthy adults. Crossover design, allocated into either passive stretching or control condition. Men with at least five years of consistent participation in aerobic exercise (4 times a week)	Interventions: Passive stretching and non-intervened CG. Muscle(s): Upper and lower body muscles. IG protocol: 3 × 30 s passive stretching (bilateral: standing pectoral stretch and standing levator scapulae arm stretch, unilateral: hamstring stretch, quadriceps stretch, calf stretch and standing posterior shoulder capsule stretch). CG protocol: No intervention.	Heart rate variability and respired gas analysis.	Root mean square of successive differences between adjacent normal RR intervals (rMSSD) (ms2): IG: 51.82 ± 26.37 CG: 52.07 ± 26.05. Mean of all normal RR intervals during 5-min recording (RR) (ms): IG: 989.8 ± 85.94 CG: 1002 ± 82.78 RR (maximal exercise test): IG: 367.9 ± 10.20 CG: 433.2 ± 29.32. Low-frequency component (ms2): IG: 3.20 ± 1.14 CG: 24.65 ± 7.57 Total power (ms2): IG: 23.57 ± 7.4 CG: 195.90 ± 46.37. VO_2max_ (ml/kg/min): IG: 55.37 ± 3.54 CG: 57.70 ± 3.94. Ve_max_ (L/min): IG: 126.9 ± 19.85 CG: 136.3 ± 19.60 RR maximum (breaths/min-1): IG: 49.08 ± 8.45 CG: 49.30 ± 8.84 Anaerobic threshold (ml/kg/min-1): IG: 25.7 ± 1.1 CG: 28.8 ± 0.8.
Mojock et al. [41]	*n* = 12 (f: *n* = 12), age: 30 ± 9 yrs., height: 159.4 ± 7.4 cm, mass: 54.8 ± 7.2 kg. Healthy adults. Crossover design, allocated into either static stretching or control condition. Trained female runners with a training volume of at least 20 km per week for at least 6 months.	Interventions: Static stretching and non-intervened CC. Muscle(s): No information. IC protocol: 18 min of static stretching (4 repetitions for each of the 5 exercises) (sit and reach, quadriceps stretch, calf stretch, lunge, gluteus stretching). CC: No intervention.	Maximal oxygen uptake, preload determination, sit and reach, preload and performance runs.	Sit and reach (cm): IG: Pre: 29.8 ± 8.6, Post: 33.1 ± 8.1 Preload run variables: HR (bpm): IC: 160 ± 12, CC: 157 ± 10 RPE (Borg scale): IC: 12 ± 1, CC: 12 ± 2 Energy (kcal): IC: 270 ± 41, CC: 270 ± 41 65% VO_2_ (ml/kg/min): IC: 33.8 ± 2.3, CC: 33.7 ± 3.1 Performance run variables: Distance (km): IC: 5.52 ± 0.69, CC: 5.53 ± 0.60 Speed (km/h): IC: 11.1 ± 1.4, CC: 11.1 ± 1.2 HR_max_ (bpm): IC: 188 ± 7, CC: 187 ± 8 RPE_max_ (Borg scale): IC: 18 ± 1, CC: 18 ± 2 HR (bpm): IC: 177 ± 6, CC: 175 ± 9 RPE (Borg scale): IC: 16 ± 1, CC: 16 ± 1
Nelson et al. [55]	*n* = 32 (f: *n* = 16, m: *n* = 16), age: no information, height: no information, mass: no information. Healthy adults. Parallel group design with random allocation (2 groups each *n* = 16 (f: *n* = 8, m: *n* = 8)). Trained college students (3–5 trainings per week for at least 30 min with 70% of the maximum heart rate).	Interventions: Static stretching and non-intervened CG. Muscle(s): Major lower extremities muscles. IG protocol: 3 × 15 s static stretching (sit and reach (4 variations), head to knee (5 variations), standing half lotus position (2 variations), quadriceps stretch (2 variations) and calf stretch (2 variations)). CG protocol: No intervention. 3 times stretching per week for 10 weeks.	VO_2peak_, sit and reach score.	VO_2peak_ (ml/kg/min): IG: Pre: 47.2 ± 11.4, Post: 47.6 ± 12.1. CG: Pre: 47.1 ± 7.0, Post: 47.8 ± 6.6. Sit and reach: IG: Pretest and posttest difference + 9%. CG: No difference. RE: There were no significant differences found for the oxygen cost of running.
Pamboris et al. [34]	*n* = 12 (m: *n* = 12), age: 27.3 ± 4.4 years; height: 1.80 ± 0.05 m; mass: 70.0 ± 0.1 kg. Crossover design with random allocation into dynamic stretching or no stretching Healthy male recreational runners	Interventions: Dynamic stretching and non-intervened CG. Muscle(s): Gastrocnemius medialis (GM) and soleus (SOL). IG protocol: 3 sets of 20 repetitions with 5s rest between the sets. CG protocol: no intervention	Online gas-analysis Calculation of RE Electromyography	Physiological Measures (m/kg/min): VO_2_– y-intercept: IG: 35.30 ± 3.99 CG: 35.24 ± 5.52 VO_2_ (1st stage): IG: 38.59 ± 4.23 CG: 39.53 ± 4.29 VO_2_ (2nd stage): IG: 42.85 ± 5.36 CG: 42.95 ± 5.41 VO_2_ (3rd stage): IG: 46.52 ± 5.62 CG: 46.65 ± 5.32 VO_2_ (4th stage): IG: 48.57 ± 5.81 CG: 49.11 ± 5.50
Panasci et al. [59]	*n* = 12 (m: *n* = 12), age: 23.8 ± 2.5 yrs., height: 176 ± 5.5 cm, mass: 73.0 ± 8.9 kg. Crossover design with random allocation into dynamic stretching, static stretching or no stretching Male recreational distance runners not participating in systematic endurance trainings and with a weekly training volume of about 15 km/week	Interventions: 5 min of running at 70% VT2 + 10 min of static stretching, dynamic stretching or running at 80% VT_2_ (no stretching) Muscle(s): gluteus, hip adductors, quadriceps, hamstrings and triceps surae. IG protocol: stretches were performed 60 s per leg, in an alternate fashion with no recovery between repetitions (stretching time 10 min) CG: 15 min of running at 70% VT_2_	RE at 75% VT_2_ and 85% VT_2_ VO_2rest_, Respiratory exchange ratio (RER), HR, Ratings of perceived exertion (RPE) Time to exhaustion (TTE), Total running distance (TRD), VO_2max_, Lactate	RE session: VO_2rest_ DS: 6.89 ± 0.23 SS: 6.90 ± 0.31 CG: 6.99 ± 0.29 VO_2_ at 75% VT_2_ DS: 34.89 ± 0.95 SS: 36.70 ± 0.83 CG: 38.27 ± 1.23 VO_2_ at 85% VT_2_ DS: 39.86 ± 0.67 SS: 41.87 ± 1.08 CG: 43.29 ± 1.20 RE at 75% VT_2_ DS: 27.36 ± 0.95 SS: 29.8 ± 0.94 CG: 31.28 ± 1.29 RE at 85% VT_2_ DS: 32.91 ± 0.69 SS: 34.97 ± 1.18 CG: 36.20 ± 1.24
Wilson et al. [56]	*n* = 10 (m: *n* = 10), age: 25 ± 7 yrs., height: no information, mass: no information, VO_2max_: 63.8 ± 2.8 ml/kg/min, body fat percentage: 6.9 ± 2.0%. Healthy adults. Crossover design with random allocation into static stretching or control condition. Trained middle- and long-distance runners with a minimum training average of 20 miles/week, and recent (≤ 3 months) participation in a competitive endurance running event (> 5 km).	Interventions: Static stretching and non-intervened CG. Muscle(s): Five muscle groups of the lower extremities (Hip extensors, Hip flexors, Plantar flexors, Knee extensors, gluteus maximus) IG protocol: 30-second repetitions of each of the 5 stretching exercises (average total stretching time 16 min) CG protocol: No intervention.	Endurance Running Performance in km, Flexibility: Sit-and-reach, Energy cost in kcal, Heart rate in bpm, Ratings of Perceived Exertion (RPE)	Distance covered during a 30-minute run as fast as possible: IG: 5.8 ± 1.0 km CG: 6.0 ± 1.1 km Sit-and-reach: IG: Before SS 24.7 ± 14.6 cm IG: After SS: 27.2 ± 14.6 cm CG: Before rest: 25.2 ± 14.6 CG: After rest: 25.5 ± 14.6 cm Energy cost (SS of IG or Rest of CG included): IG:425 ± 50 kcals CG: 405 ± 50 kcals Heart rate (bpm) peak During preload run: IG: 170 ± 5 CG: 167 ± 6 During performance run: IG: 193 ± 2 CG: 188 ± 4 RPE peak: During preload run: IG: 12 ± 1 CG: 12 ± 1 During performance run: RPE peak: IG: 18 ± 1 CG: 17 ± 1
Yamaguchi et al. [47]	*n* = 7 (m: *n* = 7), age: 21.3 ± 2.1 yrs, height: 170.3 ± 3.1 cm, mass: 60.0 ± 5.5 kg, VO_2max_: 4.35 ± 0.53 L·min⁻¹. Healthy adults. Crossover design with random allocation into dynamic stretching or control condition. Trained middle- or long-distance runners.	Interventions: Dynamic stretching and non-intervened CG Muscle(s): Five muscle groups of the lower extremities (Hip extensors, Hip flexors, Leg extensors, Leg flexors, Plantar flexors) IG protocol: DS: One set of 10 repetitions of each stretching synchronized with the tempo of a digital metronome at 30 bpm. DS was performed as quickly as possible without bouncing (total duration of DS was 3:45 min on average). CG protocol: No intervention.	Total running distance (TRD) during running at a velocity equivalent to 90% of VO2 until exhaustion, endurance running performance in time to exhaustion (TTE), Physiological and metabolic parameters in VO2, Rating of perceived exertion (RPE), Heart rate in bpm, Lactate	TRD (Distance until exhaustion): IG: 4,301.2 ± 893.8 m CG: 3,619.9 ± 783.3 m TTE: IG: 928.6 ± 215.0 s CG: 785.3 ± 206.2 VO_2_: Rest: IG: 0.21 ± 0.14 CG: 0.12 ± 0.12 Exhaustion: IG: 4.23 ± 0.56 CG: 4.27 ± 0.57 Lactate (mM x L⁻¹) Rest: IG: 1.07 ± 0.18 CG: 1.04 ± 0.33 Exhaustion: IG: 6.67 ± 1.79 CG: 6.11 ± 1.59 Heart rate (bpm) Rest: IG: 73.3 ± 7.3 CG: 71.3 ± 5.4 Exhaustion: IG: 185.6 ± 6.2 CG: 185.4 ± 9.7
Yamaguchi et al. [33]	*n* = 8 (m: *n* = 8), age: 19.9 ± 1.1 yrs., height: 171.1 ± 6.5 cm, mass: 59.4 ± 4.1 kg, VO_2max_ 4.22 ± 0.33 L·min^− 1^. Healthy adults. Crossover design with random allocation into dynamic stretching or control condition. Trained long-distance runners.	Interventions: Dynamic stretching and non-intervened CG (Each participant performed a general warm-up with running for 15 min on the treadmill at a velocity (13.37 ± 1.56 km·hr − 1) equivalent to 70% VO_2max_) Muscle(s): Five muscle groups of the lower extremities (Hip extensors, Hip flexors, Leg extensors, Leg flexors, Plantar flexors) IG protocol: DS: One set of 10 repetitions of each stretching synchronized with the tempo of a digital metronome at 30 bpm. DS was performed as quickly as possible without bouncing (total duration of DS was 3:45 min on average).	Endurance running performance in time to exhaustion (TTE), Physiological and metabolic parameters in VO2, Rating of perceived exertion (RPE), Heart rate in bpm	TTE: IG: 640.6 ± 220.4 s CG: 760.6 ± 249.1 s VO2: During GWU: IG: 2.84 ± 0.23 CG: 2.86 ± 0.20 During Performance Test: IG: 3.66 ± 0.36 CG: 3.76 ± 0.29 At Exhaustion: IG: 3.93 ± 0.40 CG: 4.09 ± 0.36 Heart rate in bpm: During GWU: IG: 146.5 ± 13.3 CG: 145.9 ± 12.7 During Performance Test: IG: 173.6 ± 11.5 CG: 178.4 ± 7.5 At Exhaustion: IG: 185.4 ± 10.9 CG: 186.3 ± 9.0 RPE: Rest: IG: 6.8 ± 1.3 CG: 6.6 ± 1.0 Post GWU: IG: 11.3 ± 1.0 CG: 11.5 ± 1.4 At Exhaustion: IG: 17.9 ± 1.2 CG: 18.1 ± 1.5
Yamaguchi et al. [58]	*n* = 16 (m: *n* = 16), age: 20.9 ± 2.1 yrs., height: 171.6 ± 3.6 cm, mass: 61.4 ± 5.7 kg. Healthy adults. Crossover design with random allocation into running intervention or dynamic stretching. Trained long-distance runners with a training volume of 4–6 times per week.	Interventions: IG 1: DS + Rest IG 2: Running + Rest Muscle(s): Five muscle groups of the lower extremities (Hip extensors, Hip flexors, Leg extensors, Leg flexors, Plantar flexors) IG Protocol: IG 1: One set of 10 repetitions of each stretching synchronized with the tempo of a digital metronome at 30 bpm. DS was performed as quickly as possible without bouncing. (total duration of DS was 3:40 min on average.) IG 2: Running: Participants ran on a treadmill at a velocity equivalent to 70% of their maximal oxygen uptake (VO_2max_) for 15 min.	Endurance running performance in time to exhaustion (TTE), Physiological and metabolic parameters in VO2	No numerical data provided (just three figures, no tables, no data in the text) TTE: The time to exhaustion after dynamic stretching VO_2_: change in average VO_2_ after both interventions showed a significant interaction
Yamaguchi et al. [57]	*n* = 8 (m: *n* = 8), age: 20.5 ± 1.1 yrs., height: 171.8 ± 2.9 cm, mass: 62.3 ± 3.2 kg. Healthy adults. Crossover design with random allocation into dynamic stretching + 5 min rest, dynamic stretching + 10 min rest or control condition. Trained middle- or long-distance runners with a training volume of 4–6 times per week.	Interventions: IG 1: DS + 5 min rest IG 2: DS + 10 min rest CG: No intervention. (All groups performed a general warm up at a velocity equivalent to 70% of VO_2max_ for 15 min) Muscle(s): Five muscle groups of the lower extremities (Hip extensors, Hip flexors, Leg extensors, Leg flexors, Plantar flexors) IG protocol: DS: One set of 10 repetitions of each stretching synchronized with the tempo of a digital metronome at 30 bpm. DS was performed as quickly as possible without bouncing. (Movements: hip flexors, hip extensors, knee extensors, knee flexors, and ankle plantar flexors) CG protocol: No intervention.	Endurance running performance in time to exhaustion (TTE), Physiological and metabolic parameters in VO2, Rating of perceived exertion (RPE)	TTE: IG 1 (5 min rest): 734.5 ± 274.2 s IG 2 (10 min rest): 884.5 ± 244.9 s CG: 719.8 ± 227.9 s VO2: During Performance Test: IG 1: 3.65 ± 0.51 IG 2: 3.81 ± 0.59 CG: 3.62 ± 0.56 At Exhaustion: IG 1: 3.86 ± 0.59 IG 2: 4.07 ± 0.78 CG: 3.81 ± 0.65 RPE: Rest (Before Performance Test): IG 1: 8.4 ± 2.3 IG 2: 8.3 ± 2.4 CG: 7.8 ± 1.8 Pre-performance Test (Approximately One Minute Before): IG 1:10.9 ± 1.7 IG 2:10.6 ± 1.7 CG: 12.3 ± 1.4 At Exhaustion: IG 1:17.6 ± 1.0 IG 2:18.0 ± 1.4 CG: 17.8 ± 1.2
Zourdos et al. [35]	*n* = 14 (m: *n* = 18), age: 23.0 ± 4.3 yrs, height: no information, weight: no information, body fat: 7 ± 2%. Healthy adults. Crossover design with random allocation into either dynamic stretching or control condition. Trained male runners with a training volume of at least 20 miles per week, a V̇O2 max ≥ 55 ml/kg/min, a recent (≤ 3 months) participation in a competitive running endurance event (> 5 km), at least 3 years of competition experience.	Interventions: Dynamic stretching and non-intervened CG. Muscle(s): Quadriceps, hamstrings, calves, and hip extensors and flexors. IG protocol: 15 min dynamic stretching (2 sets of 4 repetitions of each of the 10 movements) CG protocol: No intervention	VO_2max_, VO_2rest_, HR, sit and reach, rating of perceived exertion (RPE), distance covered in 30-minute time trial, energy cost of running at 65% VO_2max_ for 30 min	VO_2rest_ IG: Prestretch: 6.2 ± 1.7 Poststretch: 8.4 ± 2.1 ml/kg/min CG: Prestretch: 5.8 ± 1.1 Poststretch: 6.1 ± 1.0 ml/kg/min HR preload run (peak) G: 162 ± 18 CG: 168 ± 18 bpm HR performance run (peak) IG: 189 ± 8 CG: 189 ± 9 bpm Sit and reach IG: 32.3 ± 8.6 to ± 37.6 ± 8.1 cm CG: 32.5 ± 8.1 to 34.0 ± 8.1 cm Rating of perceived exertion (RPE) Preload run (peak): IG: 11 ± 3 CG: 12 ± 3 Performance run (peak): IG: 18 ± 1 CG: 18 ± 1 Distance covered IG: 6.1 ± 1.3 CG: 6.3 ± 1.1 km Energy cost IG: 416.3 ± 44.9 kcal CG: 399.3 ± 50.4 kcal

**Abbreviations**: **IG** = Intervention Group, **CG** = Control Group, **VO**_**2**_ = Oxygen uptake (ml/kg/min), **EE** = Energy expenditure (kJ/min or kcal/kg/min), **VE** = Ventilation (l/min), **RER** = Respiratory exchange ratio, **HR** = Heart rate (bpm), **iEMG** = Integrated electromyography, **iEMG**_**VM**_ = Integrated electromyography of vastus medialis (µV), **iEMG**_GA_ = Integrated electromyography of gastrocnemius lateralis (µV), **iEMG**_**BF**_ = Integrated electromyography of biceps femoris (µV), **La** = Blood lactate concentration (mmol/l), **ROM** = Range of motion (°), **TTE** = Time to exhaustion (seconds), **TRD** = Total running distance (m), **GWU** = General warm-up, **DS** = Dynamic stretching, **PNF** = Proprioceptive neuromuscular facilitation, **RPE** = Rating of perceived exertion (Borg scale), **VO**_**2**_**max** = Maximal oxygen uptake (ml/kg/min), **VEmax** = Maximal ventilation (l/min), **RR** = Respiratory rate (breaths/min), **rMSSD** = Root mean square of successive differences between adjacent normal RR intervals (ms^2^), **PSA** = Post-stretching dynamic activities

#### Methodological study quality and risk of bias

The study quality was rated using the “Physical Evidence Database Scale” (PEDro) for the assessment of methodological study quality [43, 44]. Scoring was performed by two independent investigators (MZ and SDS). If consensus was not reached, a third examiner provided the decisive vote (KW).

#### Data processing and statistics

For each outcome of interest, the mean and standard deviation were extracted by MZ and transferred to a separate Excel sheet, while LB and SDS double-checked the results. If studies reported other than means with standard deviations, the required metrics were calculated to include the studies in our analysis. ES were calculated using the post-test values from the intervention and control groups to determine mean differences and pooled standard deviations. Therefore, posttest only designs were included due to study procedure heterogeneity: Several studies did not include pre-posttest design as the pre-test (running) was performed on a separate occasion. Therefore, effect sizes were calculated based on post-test comparisons. Most of the included studies determined the VO_2max_ on a separate day whereas RE assessment protocols varied depending on the study design.

To account for multiple dependent study outcomes and the heteroscedasticity of the data distribution, Fisher and Tipton suggested using the robust variance estimation (RVE) model to pool standardized mean differences (SMDs) and 95% confidence intervals (CIs) between the intervention and control groups [45]. The between-study variance component was estimated using τ^2^, while the intra-study heterogeneity was quantified using Ω^2^. Pooled ES were interpreted as follows: 0 ≤ ES < 0.2 = trivial, 0.2 ≤ ES < 0.5 = small, 0.5 ≤ ES < 0.8 = moderate, and ES ≥ 0.8 = large [46]. In addition to the omnibus analyses on the overall effects of stretching, we performed subgroup analyses for stretching types, as it was speculated that different stretching types could cause different adaptations [47], while methodological heterogeneity was accounted for by sensitivity analyses, if applicable. All the calculations and graphical illustration were performed using R with the robumeta package [48].

The risk of publication bias was assessed using funnel plots that were modified to account for dependent multiple-study outcomes [49].

#### Certainty of evidence

The certainty of evidence was classified in adherence to the “Grading of Recommendations, Assessment, Development and Evaluation” (GRADE) working group criteria [50]. Starting with the assumption of a high quality of evidence when considering randomized controlled trials, the quality of evidence was adjusted considering the provided criteria. In detail, limitations in study design or execution, inconsistency of results, indirectness of evidence, imprecision or publication bias would each cause one point reduction until the certainty of evidence could be graded as very low. On the contrary, for example, large magnitude effects or a dose-response gradient were criteria that upgraded the certainty level.

## Results

### Study selection

**Fig. 1:**
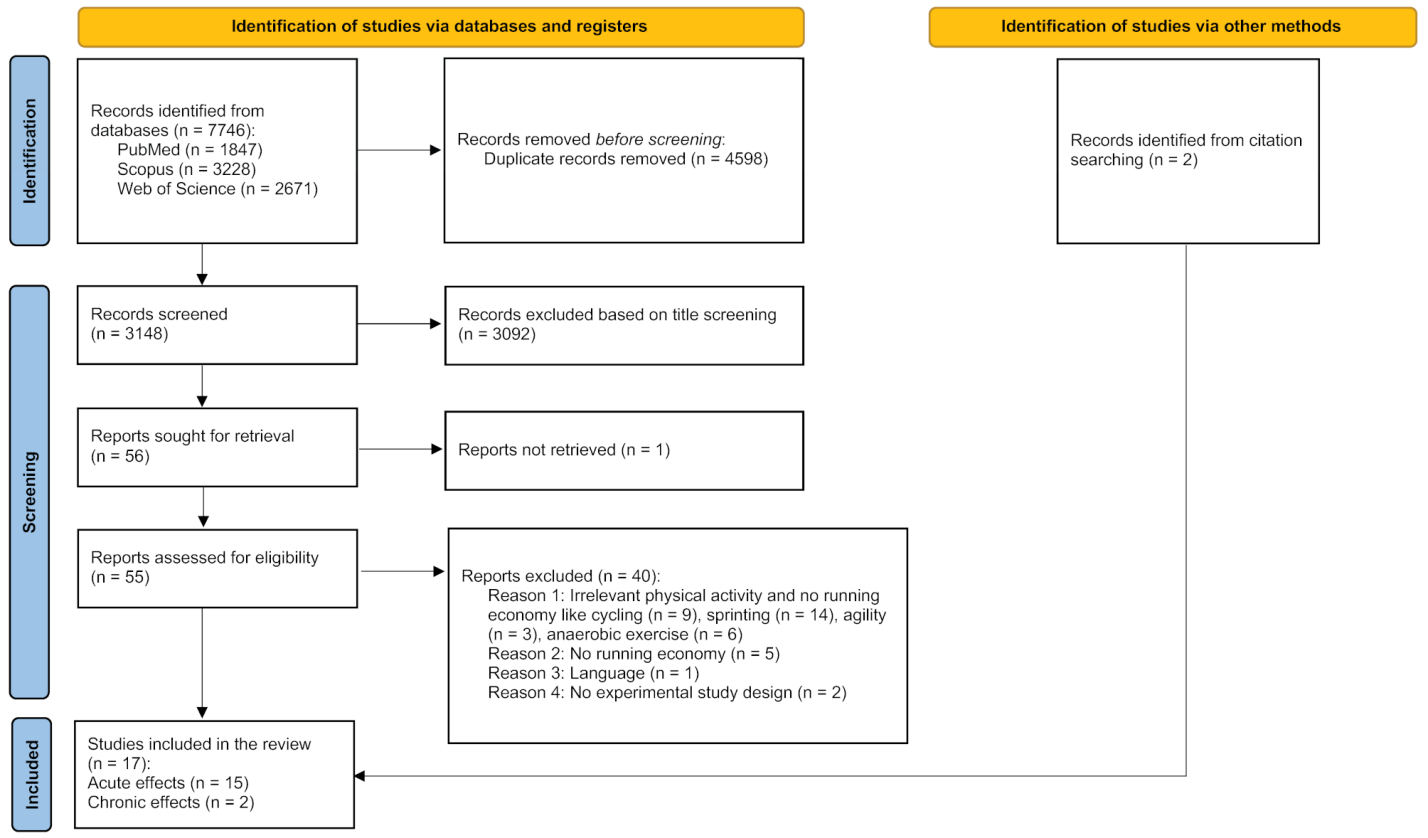
Flowchart of the literature search for the effects of stretching on RE [60]. For more information, visit: http://www.prisma-statement.org/)

Collectively, the search terms retrieved 7746 hits from the three databases. After title, abstract, and full text screening based on the inclusion and exclusion criteria, a total of 17 eligible studies [31–35, 41, 42, 47, 51–59] for the systematic review were identified (Fig. 1).

### Study characteristics

While two studies [47, 53] included seven participants, the maximum sample size was recruited by Godges et al. [52], with 25. The training status within studies was heterogeneous, with studies including healthy “recreationally trained” participants [32, 34, 42, 52, 59]. Nomenclatures from original research were adopted and summarized as recreationally trained ranging from “recreational male runners with a training volume of about 15 km/week” [32] to “Men with at least five years of consistent participation in aerobic exercise (4 times a week)” [31].

Allison et al. [51], Hayes & Walker [53], Konrad et al. [54], Maior et al. [31], Mojock et al. [41], Nelson et al. [55], Wilson et al. [56], all the Yamaguchi et al. studies [33, 47, 57, 58], and Zourdos et al. [35] investigated the effects of stretching on RE in “well-trained endurance athletes”. This included groups like “Competitive male middle and long-distance runners with a training volume about 68 km per week” [53] or “Trained male runners with a training volume of at least 20 miles per week, a V̇O2 max ≥ 55 ml/kg/min, a recent (≤ 3 months) participation in a competitive running endurance event (> 5 km), at least 3 years of competition experience” [35].

### Methodological quality

On average, the methodological quality of the included studies was rated as fair [61] (mean 4.88 out of 10 points; range 4 to 6 points; see Table S1 in the Supplementary Material) assessed by PEDro. All studies performed random group allocation (17/17), while in no study was the allocation concealed (0/17). 35% of the studies reported that the investigated groups were similar at baseline (6/17). Blinding was never achieved, neither for participants (0/17), nor therapists (0/17), nor assessors (0/17). Only half of the studies specifically stated or showed in their data that at least 85% of the initially included subjects were analyzed (8/17). All studies adhered to the principle of “intention to treat” (17/17). All reported statistical between-group comparisons (17/17) and all provided point measures and measures of variability (17/17).

### Risk of publication bias

**Fig. 2:**
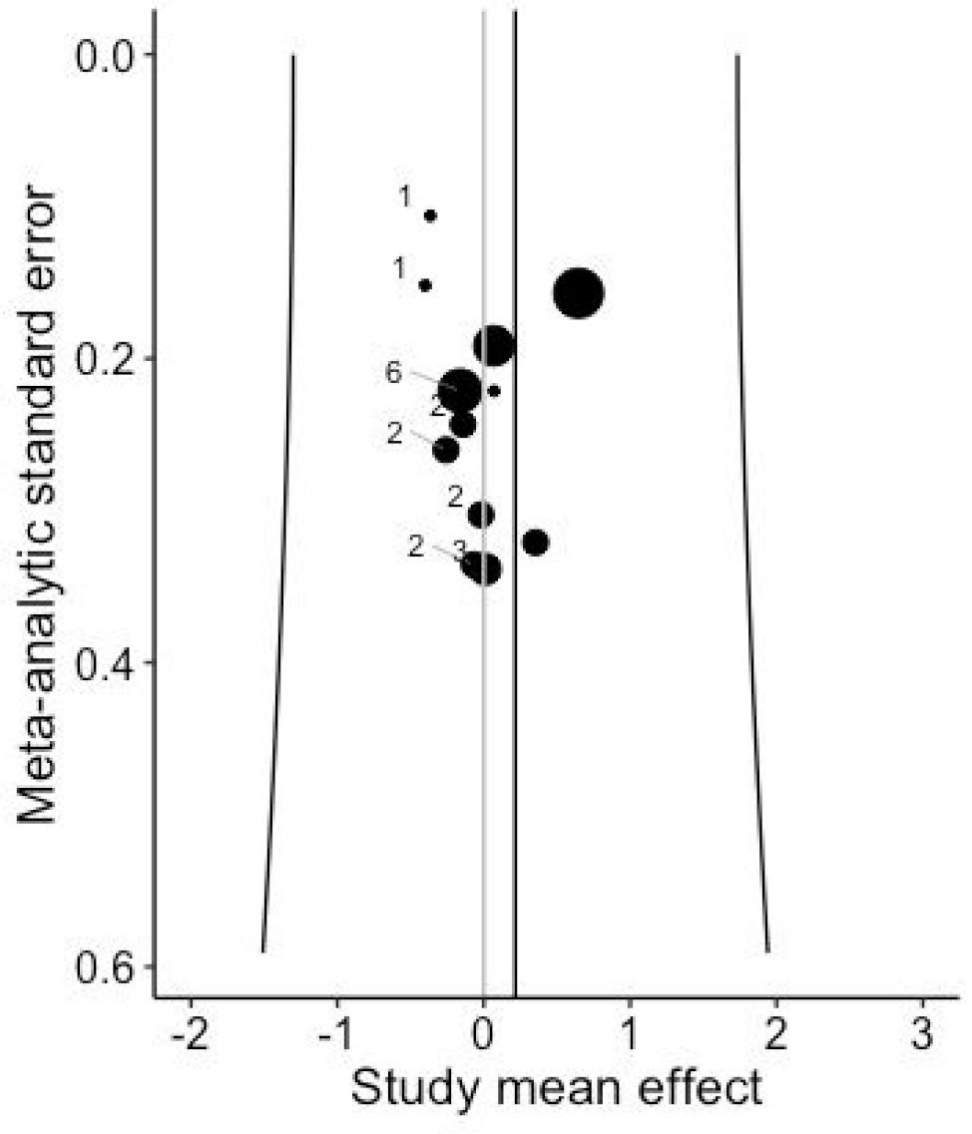
Funnel plot accounting for multiple intra-study effects for visual inspection of the publication bias, with the numbers near the circles showing the included effect sizes within a cluster

The visual inspection indicated no risk of publication bias (Fig. 2).

### Qualitative study results

#### Participant characteristics

**Fig. 3:**
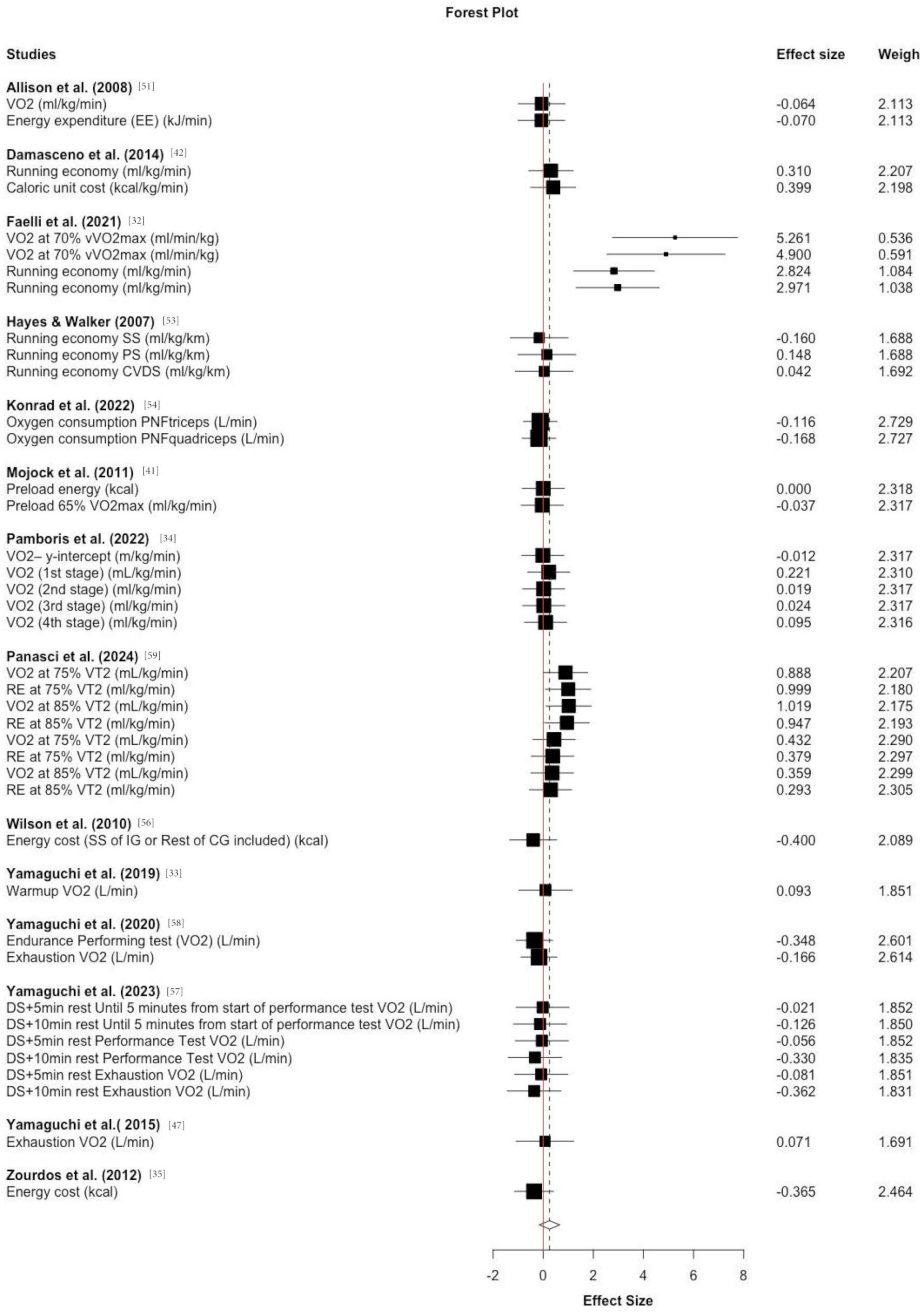
Forest plot for overall acute effects for static stretching (SS), dynamic stretching (DS), proprioceptive neuromuscular facilitation stretching (PNF), and prolonged stretching (PS) on running economy (RE) or oxygen consumption (VO_2_), at prescribed velocities, ranging between 65% VO_2max_ to 90% VO_2max_, or as fixed velocities, as described by the authors

A total of 16 studies were evaluated, with 14 studies addressing acute effects in 181 (169 m, 12f) participants (age: 26.06 ± 5.3 years (one study did not provide detailed participant characteristics [41])), while two investigated chronic adaptations in 57 (41 m, 16f) participants (age: 21 years; neither study provided detailed participant characteristics) [52, 55] (Table 1). Overall, the qualitative study results are illustrated in Fig. 3.

#### Stretching intervention

Out of 15 studies directly exploring the effects of stretching on RE, six used exclusively static stretching [31, 41, 42, 51, 52, 55], while in another six studies only dynamic stretching [33–35, 53, 57, 58] was used [33, 34, 47, 54, 57] with one using PNF stretching [54]. In three additional studies, both static and dynamic stretching were utilized [32, 53, 59].

In the static stretching studies, the total stretching duration per session ranged from 150 s [32] to 960 s [51] and was applied with a minimum of one [32] to four repetitions [41, 56].

The time required for each repetition varied between 15s [55] and 60 s [51, 59], with an inter-set rest of between 0 [31, 59] to 15 s [51]. Six studies were unable to find any effect of the stretching intervention on RE, with *p*-values of between > 0.72 [51] and 0.943 [53].

Dynamic/PNF stretching interventions ranged between 36 s [34] and 300 s [53], with the number of repetitions varying from one [35, 59] to 20 [34]. The only studies to provide a clear time for the inter-set rest was Pamboris et al. [34], namely 5 s, while there was no inter-set rest in the study of Panasci et al. [59]. Studies showed “negative to positive” effect sizes ranging from − 0.362 [57] to 0.789 [32], indicating detrimental to improving effects on RE [32], with only a small number of studies indicating positive influence of stretching on RE [32, 59].

Nearly all the studies conducted the intervention in all the lower extremity muscles. Only in the study by Konrad et al. [54] did the intervention group engage in stretching exercises targeting either the triceps surae or the quadriceps muscles, while Pamboris et al. [34] exclusively stretched the gastrocnemius and soleus muscles.

#### Running economy

The running protocols used to assess RE ranged from 40 to 90% of VO_2max_ over measurement durations from 30 s to 30 min and running speeds from 2.3 m/s to 17.36 km/h. Several studies used intensities between 65% [35, 41, 56] and 70% VO_2max_ [32, 51, 54, 55] for 30 min and 5 min measurement durations, respectively. Other protocols used were 3 min incremental step tests, with RE averaged over the last 30 s of each step [34] or at VO_2max_ and the anaerobic threshold [31], while Yamaguchi et al. had subjects run at 90% VO_2max_ until exhaustion [33, 57, 58]. Panasci et al. tested the VO_2_ and RE at 75% and 85% of VT2 [59].

#### Energy consumption

The reporting of energy consumption varied across the studies. Allison et al. [51] quantified energy expenditure in kilojoules per minute (kJ/min), Mojock et al. [41] measured energy in kilocalories (kcal), and Damasceno et al. [42] identified the caloric unit cost in kilocalorie per kilogram bodyweight per minute (kcal/kg/min). Two additional studies quantified energy cost in the form of kcal [35, 56].

In the static stretching studies, only Damasceno et al. [42] and Allison et al. [51] provided information on the stretching time, which ranged from 630 to 960 s. The time per repetition ranged from 30 s [42] to 40 s [51].

The effect sizes ranged from − 0.399 [42] to 0.4 [56], while only Wilson et al. [56] found a significant effect (*p* < 0.05), indicating increased energy costs after dynamic stretching.

The study by Zourdos et al. [35] indicated a detrimental dynamic effect of stretching on energy expenditure (d = 0.356, *p* < 0.05).

The general effectivity of stretching was measured via ROM testing [35, 41, 51], which showed significant increases (*p* = 0.001 to 0.05).

### Quantitative analysis of acute effects

**Fig. 4:**
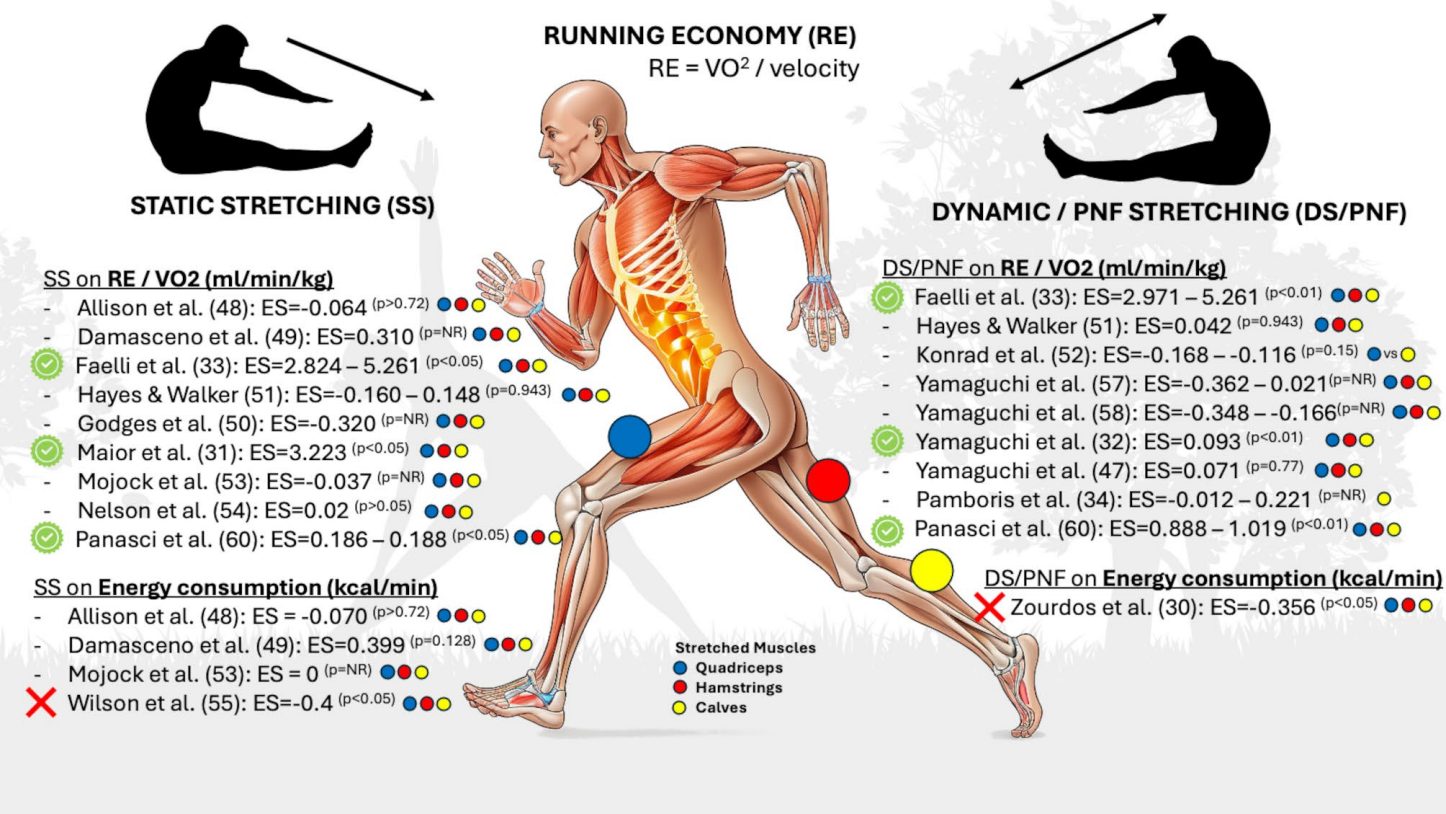
Graphical summary of the qualitative effects of stretching on running economy (RE) and energy consumption (EC), with *p*-values cited from the original studies, effect sizes calculated via robust variance estimation analysis, and colors symbolizing the muscles targeted by the stretching program within the intervention session. Green tick = improvement in RE or EC, - = no significant effect on RE or EC, red cross = negative effect on RE or EC

The 15 included studies provided 40 effect sizes that could be considered for the meta-analytical effect calculation. Of these studies, six performed static stretching for 15 to 120 s, while nine studies performed dynamic or PNF stretching. Neither the overall analysis (ES = 0.25, − 0.16 to 0.66 [95% CI], *p* = 0.21, τ^2^ = 0.25, Ω^2^ = 0, see Fig. 4) nor the subgroups for static (ES = 0.33, − 0.25 to 0.92 [95% CI], *p* = 0.21, τ^2^ = 0.31, Ω^2^ = 0, seven studies and 15 effect sizes) or dynamic (ES = 0.21, − 0.27 to 0.69 [95% CI], *p* = 0.34, τ^2^ = 0.24, Ω^2^ = 0, ten studies and 25 effect sizes) stretching reported any significant acute effect of stretching on RE. Due to methodological heterogeneity, a sensitivity analysis was performed excluding Faelli et al. [32] and Maior et al. [31] for subgroup analyses as one study showed either larger effects compared to all the other studies [31], and the other only provided interquartile values instead of common standard deviations or errors [32] (probably stemming from a lack of normal distribution of data) which only allows estimates of the standard deviation. Nevertheless, this did not significantly affect the effect size classification/significance classification (static: ES = 0.14, − 0.15 to 0.43 [95% CI], *p* = 0.28, τ^2^ = 0, Ω^2^ = 0, dynamic: ES = 0.08, − 0.32 to 0.49 [95% CI], *p* = 0.65, τ^2^ = 0, Ω^2^ = 0. Therefore, the hypothesized outlier had no statistically relevant effect here. Graphical summary of the qualitative effects of stretching on running economy (RE) and energy consumption (EC), with *p*-values cited from the original studies, effect sizes calculated via robust variance estimation analysis, and colors symbolizing the muscles targeted by the stretching program within the intervention session. Green tick = improvement in RE or EC, - = no significant effect on RE or EC, red cross = negative effect on RE or EC.

### Qualitative analysis of chronic effects

As only two studies, with each providing one effect size, were found that investigated the chronic effects of stretching on RE, no quantitative analysis was performed. Both studies were performed more than 20 years ago [52, 55] and explored the effects of three weeks and ten weeks of static stretching on RE at velocities corresponding to 40% and 80% VO_2max_ [52] and 70% VO_2max_. Interestingly, while Godges et al. [52] hypothesized a positive influence of static stretching (two times per week for 6 min on each thigh), Nelson et al. [55] suggested that stretching could impair RE. Nevertheless, in both groups, the authors reported no significant effects, positive or negative.

#### Certainty of evidence

Considering the GRADE criteria, the certainty of evidence was initially rated as high due to the inclusion of (randomized) controlled trials on acute stretching and running economy (RE). However, the level of evidence was finally graded as very low, with a three level downgrade primarily due to risk of bias (lack of blinding and allocation concealment, fair to poor PEDro scores), risk of publication bias (indicated by Fig. 2 and Egger’s test) and large heterogeneity for the overall calculation. Although the meta-analysis suggests no significant acute influence of stretching on RE, the overall confidence in this null finding remains limited.

For the two studies examining chronic stretching interventions (3 to 10 weeks) on RE, the certainty of evidence was likewise initially rated as high but was ultimately downgraded to very low. This reflects major concerns about risk of bias, small sample sizes, and insufficient reporting—no allocation concealment or blinding was reported, and both trials were conducted over two decades ago. Consequently, the available data do not allow any firm conclusions regarding a long-term effect of stretching on RE.

## Discussion

In several articles, RE was highlighted as a crucial determinant for success in middle- and long-distance runners [4, 62, 63]. Comparable to the stretch-induced force deficit [38, 64, 65], recommendations to avoid stretching before running lack evidence. Due to the low quality and methodological issues, drawing final conclusions about the effects of stretching on RE based on the available studies seems problematic. Nevertheless, without reaching the level of significance, the current evidence does not support endorsements to avoid stretching prior to running, and there are no current studies exploring chronic effects.

In the existing literature, it is assumed that a stiffer MTU has a beneficial influence on energy storage and release capacity [12], with an assumed influence on RE. A stiffer tendon facilitates the rapid return of stored elastic energy during the stretch-shortening cycle, potentially reducing the metabolic cost of running by decreasing the reliance on active muscle contractions. Conversely, a more compliant tendon may enable the muscle to operate closer to an isometric state, which can also enhance efficiency by minimizing energy expenditure for active force generation. The optimal balance between tendon stiffness and compliance likely depends on factors such as running speed, ground reaction forces, and neuromuscular coordination [18, 66]. Several articles applied different interventions to affect the stiffness of the respective tissue and showed that increased stiffness was related to improved RE [12, 13]. Meanwhile, a large body of evidence supports the beneficial effects of resistance training on RE [19, 20], which has also been attributed to increased stiffness [67], while well-designed stretching studies are scarce.

Heavy resistance training and jump training/plyometrics indicated significant effects on oxygen consumption and RE (resistance training: ES = − 0.426 to − 0.32, *p* = 0.018 [20, 21]) (jump- and plyometrics: ES = − 0.307 to 0.55, *p* = 0.012 to 0.028) [20, 68]. In contrast, submaximal (ES = − 0.365, *p* = 0.131) and isometric resistance training (ES = − 0.269, *p* = 0.253) have been reported as having no effect on RE [20, 69]. Concerning stiffness as a potential underlying parameter of RE [70], while concurrent strength and endurance training have been reported to have no effect on triceps surae stiffness (ES = 0.60, *p* > 0.05) [71], a meta-analysis showed that plyometrics increases lower limb stiffness (ES = 0.33, *p* = 0.01) [72]. In this manner, a meta-regression revealed that stiffness increased with the strain intensity of the resistance training protocol [22]. Unfortunately, due to the study design heterogeneity, no conclusive statement on the impact of stretching intensity on RE can be provided as a result of this analysis, avoiding conclusive statements regarding underlying mechanisms.

### Acute effects of stretching

Stretching is a known moderator of flexibility and stiffness [23, 73]. Consequently, it has been hypothesized that static stretching of the muscle-tendon complex prior to running in training or competition would impair RE [74], especially for shorter distances, as the detrimental effects seem to be limited to the first minutes of an endurance exercise [75].

The literature provides inconclusive and controversial evidence on stretching effects on RE. These conflicting results are reflected in the results of this review, showing neither significant overall effects for all types of stretching (*p* = 0.21), nor there was any stretching type subgroup effect (0.21–0.34). In the following sections, several issues are discussed that might explain these results. The comparatively large heterogeneity and 95% CIs should be noted, calling for careful results interpretation. This problem could not be solved by performing sensitivity analyses for larger effects in both subgroup analyses. Only Maior et al. [31], Panasci et al. [59], Faelli et al. [32] and Yamaguchi et al. [33] indicated significant positive effects of stretching on RE, while all remaining studies had no significant influence on the RE.

### Muscle, tendon and muscle-tendon-stiffness as a potential moderator of RE

Assuming that stretching would alter the plantar flexor/Achilles tendon complex stiffness, which would, in turn, decrease energy storage and release capacity [12], this effect could negatively impact RE. In contrast, Pamboris et al. [34] described that a more compliant tendon would store even more elastic energy [76], and a stiffer tendon and aponeurosis would enhance the energy consumption during the propulsion phase in running tasks. We refer to a modeling study by Ettema [77], who proposed that a more compliant MTU would optimize the storage and release of series elastic energy. This suggestion is in line with the results from Kubo et al. [78], who argued that increased ground contact times would allow a greater percentage release of stored energy within the tendinous tissue [12]. Arampatzis et al. [13] suggested that a more compliant MTU could slow down the rate of muscle contraction. According to the force-velocity curve of a muscle, as noted by Hill [79], a muscle can thus generate more force, which in turn could lead to less muscle activity being needed for the same force production. Therefore, while there is consensus that high metabolic cost dominantly determines the required energy in locomotion [80, 81], and thus depends on the muscle activity, there is a discrepancy in the underlying assumption of whether a tendon should be stiff, or not. Moreover, it seems that stiffness requirements could be body segment specific, meaning that high Achilles tendon stiffness could be beneficial, while this does not necessarily apply to the muscle-tendon properties of the upper thigh muscles. Indeed, Arampatzis et al. [13] and Bohm et al. [82] showed higher compliance in the quadriceps tendon and aponeurosis to positively impact RE, while the same author group simultaneously found that a stiffer Achilles tendon could also provide benefits. Since almost all the included studies used a whole lower body stretching program, Konrad et al. [54] hypothesized that positive (reduced quadriceps stiffness) and negative stretching effects (reduced Achilles tendon stiffness) could be counterbalanced, which might result in zero effect. To counteract, these authors were the only ones to use isolated quadriceps and plantar flexor PNF stretching (4 × 15 s). However, none of the interventions significantly influenced RE.

### Lack of underlying mechanism evaluation in stretching studies on running economy

Since almost all of the included studies in the review hypothesized that RE would be affected by muscle/tendon/MTU stiffness, it seems highly surprising that none of the included studies actually measured stiffness parameters. In addition, the study rationale derivation seems biased by unbalanced stretching results presentation [41, 42, 56], as they focused on studies that showed significant stretching effects on stiffness, while neglecting study results showing that stretching did not modulate stiffness parameters. For instance, while Konrad et al. [25] showed that static, ballistic, and PNF stretching decreased the stiffness of the MTU, the effects could also be attributed to impacted nociceptive nerve endings, and thus delayed occurrence of stretching pain [83]. Krause et al. [84] also found that stretch-induced ROM enhancements were not accompanied by significant stiffness changes after conducting 2 × 60 s of static stretching, while Magnusson also cast doubt on passive property changes by attributing the effects to stretch tolerance increases [85]. Even though the most recent meta-analysis by Takeuchi et al. [24] indicated that stretching can affect MTU stiffness acutely when compared to passive controls, these effects were also reported after several concurrent exercise routines, and thus cannot be attributed exclusively to the stretching [27]. Speculative effects on RE are further complicated as MTU stiffness cannot be considered equal to tendon stiffness, which was the assumed moderator for RE [54]. Even though Konrad et al. [54, 86] stated that PNF stretching could potentially affect tendon stiffness [25, 87] in their RE study [54], they did not measure such effects. Thus, by assuming stiffness would impact RE, the conducted research designs were not valid to evaluate this hypothesis.

### Stretching duration and its impact on running economy

Crucial for this assumption might be the duration of the stretching, as only medium durations of < 90 s have been shown to improve RE [36], while longer stretch durations also correlate acutely with the magnitude of the effect on MTU stiffness (coefficient = − 0.0061, *p* = 0.011), indicating that a longer stretch duration decreases stiffness to a greater extent [23]. Significant decreases in stiffness occurred with stretching durations of > 90 s [88] and > 180 s [89, 90]. Due to this discrepancy between the effects on RE (short stretching durations) and the effects on stiffness (longer stretching durations), the effects might counterbalance each other.

Another interpretational problem might arise from a simplified approach, as almost all the studies derived their study rationale under the assumption that stretching would affect stiffness, which, in turn, would moderate RE. Even though this approach seems reasonable, simplification to one-dimensional approaches seems to neglect several further parameters in a multifactorial construct. Accordingly, in their review, Barnes and Kilding [3] identified several further biomechanical and metabolic parameters that could influence RE. Out of many, another parameter that has been frequently addressed is neuromuscular efficiency.

### Confusing underlying physiology of explosive strength and running economy

It is also conceivable that a stretching intervention changes the running technique, which could further explain the improvement or deterioration in RE. Therefore, it will be necessary to include these parameters in future studies to more comprehensively understand the effects of a stretching intervention [91]. Luthanen and Komi reported contact times of < 200 ms for endurance runners [92, 93]. As strongly related to stretch-shortening cycle (SSC) activities, some authors [42, 74] have assumed that (explosive) strength would critically affect RE. Therefore, several studies [54] have referred to evidence that showed that prolonged static stretching would impair subsequent speed performance related parameters [64, 65, 94]. This assumption must be considered a misinterpretation, as (explosive) strength is predominantly determined by maximal central nervous innervation, reflected in maximal frequency of synchronous fiber recruitment [95]. If the ability to explosively/maximally exert force, and thus maximally innervate the muscle, critically determines middle- and long-distance running performance, a marathon would be, in fact, impossible. Meanwhile, for sprinters, the ability to exert maximal force within a very small time window is of crucial importance [2], due to the completely contrasting physiological mechanisms [96, 97]. Therefore, it is important to distinguish between explosive strength and most economic running. Even though, in both, tendinous properties play a role (and thus energy storage in the tendon), these parameters do not refer to explosive strength. Therefore, referring to studies that show stretch-induced impaired explosive and speed strength performance is invalid when explaining RE effects, especially since updated evidence did not confirm stretch-induced athletic performance impairments [38].

### Chronic effects of stretching

To date, there are only two studies that have investigated the effects of chronic stretching interventions with 3 to 10 weeks’ duration on RE. While 3 weeks of stretching seems insufficient to induce any structural adaptations per se [98], high stretching intensities and volumes must be implemented in future research to reasonably assume structural adaptations [99, 100], and thus potentially affect RE. In their review article, Barnes and Kilding [3] graphically illustrated the potential influencing factors on RE. Stiffness was just one factor. When aiming to investigate the influence of just one parameter in a multifactorial construct that is impacted by several factors, in the case of stretching, dosages that are enough to cause large-magnitude effects should be induced to first explore if any effects can be assumed, before implementing short stretching durations. Unfortunately, to date, no such intervention has been conducted.

### The role of training-independent parameters

“You are born as a sprinter” is still a popular belief, referring to a specific type II fiber distribution in sprinters, which might be the result of a specific gene pool [2]. However, in middle- and long-distance running athletes, there also seem to be factors which are determined by specific anatomical attributes and possible genes, which might be hardly modifiable. For instance, there have been extensive and also controversial discussions on the Achilles tendon moment arm, which seems to be moderately to highly correlated (*r* ≤ 0.75) with running speed and metabolic consumption [8, 101, 102]. Returning to the subject of Kenyan running dominance, Kunimasa et al. [103] showed that the shank and Achilles tendon architecture of elite Kenyan runners differed significantly to those of Japanese runners and attributed the specificity of the muscle-tendon and foot architecture of elite Kenyan runners to genetic factors. Accordingly, running performance was moderately determined by tendon length and muscle pennation/fiber lengths, indicating anatomical attributes that resulted in a more economical way to run [1, 103, 104].

### Methodological considerations of the included studies

As all the included studies investigated RE on a treadmill, it has to be considered that the treadmill running technique is different from running on the ground [32], as runners need to produce less propulsive force due to the lack of wind resistance on the treadmill [105], affecting VO_2_. Most of the included studies did not report if runners were familiarized [3] with treadmill running mechanics, which could limit adaptations in RE. The assessment of RE is also influenced by the different gas analysis equipment, protocol variations, and data analysis techniques [3, 70]. Furthermore, the training levels of the included runners in the investigated studies differed, with some including highly trained athletes with VO_2max_ of > 60 ml/min/kg [33, 35, 47, 51, 53, 56–58], while others used recreationally active subjects [31, 32, 34, 41, 42, 52, 54, 55, 59]. Therefore, the heterogeneity, first, in stretching techniques (different durations, lack of intensity quantification, different types of stretching) as well as lack of consensus on velocities in which RE should be measured implies future, more homogeneous study designs to provide more valid conclusions on exercise interventions on RE parameters.

### Outlook

Assuming that RE is a multifactorial construct, depending on biomechanical and metabolic parameters [3], the focus on conflicting stretching study results on tendon stiffness without distinguishing between specific leg segments (there are different stiffness requirements in different leg segments) [54] might explain the lack of clear evidence. Furthermore, even though stiffness could crucially impact RE, moderating one factor in a complex construct must show large-magnitude effects to induce meaningful and practical relevant effects. Therefore, to affect structural properties acutely and chronically, reasonable stretching durations must be induced, as morphological changes might require adequate volumes and intensities [99, 100]. This should be explored in studies with larger sample sizes, as the current literature participant numbers ranged from seven participants [53] to a maximum participant number of 25 [52]. About three years ago, Konrad et al. [36] noted that there is an increased requirement for stretching studies on RE in female participants. However, the numbers of female participants have not increased since then, underlining the need for further well-designed research. Lastly, if hypothesizing that stiffness moderates RE, controlling the intervention effects on stiffness is essential. Therefore, studies with large stretching dosages considering specific muscle-tendon requirements (Achilles tendon/quadriceps) are warranted, as the current stretching research on RE is unsatisfactory, and does not allow any final conclusion.

### Limitations

Meta-analyses assume homogeneity in their included individual study results [106]. While this fact limits the validity of meta-analytical approaches in sports and exercise science in general, almost none of the included studies used a comparable research design. Even though inter-study and intra-study heterogeneity were considered non-existent to low, these values only account for outcome heterogeneity, while not quantifying method heterogeneity. Especially since Barnes and Kilding [70] described the high effect specificity of training effects depending on the used velocity in RE determination, it is noteworthy that all the studies used different prescribed velocities, if quantified. Even though it was possible to apply a meta-regression, due to the small number of studies and effects, the results must be interpreted with caution. “Garbage in– garbage out” [107] fits very well for meta-analytical effect size pooling, so there is a paramount requirement for high-quality stretching research on RE to improve the current scientific evidence.

## Conclusions

With our review we showed that overall, stretching did not significantly affect running performance, i.e. RE acutely, while there is a substantial lack of chronic studies. Several flaws in research design prohibit conclusive statements about underlying mechanisms, as the frequently proposed main hypothesis, that plantar flexor stretching would negatively affect RE was not explored in the original articles. Almost none of the included studies investigated muscle-, tendon-, or muscle tendon unit stiffness, and thus the main hypothesis was not evaluated. Furthermore, only one study performed separated stretching of the lower leg muscles, while others intervened on whole lower extremity muscles. This is of importance as supposed negative effects from plantar flexor stretching could be counteracted by positive effects of the quadriceps (reduced stiffness was suggested to positively affect RE). Therefore, we conclude that, in contrast to resistance- and plyometric training studies, much work needs to be done in the future to provide high quality evidence on stretching effects on RE.

## Electronic Supplementary Material

Below is the link to the electronic supplementary material.

Supplementary Material 1

## Data Availability

Excel spreadsheets are available on reasonable request. No original data were collected for this research.

## Abbreviations

CI: Confidence intervals
ES: Effect size
Kcal: Kilo calories
MTU: Muscle tendon unit
PEDro: Physical Evidence Database Scale
PICO: Participants Intervention Control Outcome guideline
PNF: Proprioceptive neuromuscular facilitation
RE: Running economy
RVE: Robust variance estimation
SMD: Standardized mean differences
VO_2max_: Maximal oxygen consumption
