# Supplementary material for: Acute and Chronic Effects of Stretching on Running Economy: A Systematic Review with Meta-Analysis
Source: Sports Med Open. 2025 May 30;11:61. doi: 10.1186/s40798-025-00859-0 (PMC12122984; doi:10.1186/s40798-025-00859-0)
Supplement: Supplementary file 1 — Supplementary Material 1 [file 40798_2025_859_MOESM1_ESM.docx]

Supplementary Material

Table S1 Quality of evidence rating via the PEDro scale for the included studies

| Study | Random allocation | Allocation concealed | Similar baseline | Participant  blinding | Therapist blinding | Investigator blinding | 85% outcome measures | Intention to treat | Group comparison | Effect via M±SD | Score |
| --- | --- | --- | --- | --- | --- | --- | --- | --- | --- | --- | --- |
| Allison et al. 2008 | Y | N | Y | N | N | N | Y | Y | Y | Y | 6/10 |
| Damasceno et al. 2014 | Y | N | N | N | N | N | Y | Y | Y | Y | 5/10 |
| Faelli et al. 2021 | Y | N | N | N | N | N | Y | Y | Y | Y | 5/10 |
| Godges et al.1993 | Y | N | N | N | N | N | Y | Y | Y | Y | 5/10 |
| Hayes & Walker 2007 | Y | N | Y | N | N | N | Y | Y | Y | Y | 6/10 |
| Konrad et al. 2022 | Y | N | N | N | N | N | N | Y | Y | Y | 4/10 |
| Maior et al. 2012 | Y | N | N | N | N | N | N | Y | Y | Y | 4/10 |
| Mojock et al. 2011 | Y | N | N | N | N | N | Y | Y | Y | Y | 5/10 |
| Nelson et al. 2001 | Y | N | Y | N | N | N | N | Y | Y | Y | 5/10 |
| Pamboris et al. 2022 | Y | N | Y | N | N | N | N | Y | Y | Y | 5/10 |
| Panasci et al. 2024 | Y | N | N | N | N | N | Y | Y | Y | Y | 5/10 |
| Wilson et al. 2010 | Y | N | Y | N | N | N | Y | Y | Y | Y | 6/10 |
| Yamaguchi et al. 2015 | Y | N | N | N | N | N | N | Y | Y | Y | 4/10 |
| Yamaguchi et al. 2019 | Y | N | N | N | N | N | N | Y | Y | Y | 4/10 |
| Yamaguchi et al. 2020 | Y | N | N | N | N | N | N | Y | Y | Y | 4/10 |
| Yamaguchi et al. 2023 | Y | N | N | N | N | N | N | Y | Y | Y | 4/10 |
| Zourdos et al. 2012 | Y | N | Y | N | N | N | Y | Y | Y | Y | 6/10 |
| Overall: | | | | | | | | | | | 4.88 |
